# Metabolite release by nitrifiers facilitates metabolic interactions in the ocean

**DOI:** 10.1093/ismejo/wrae172

**Published:** 2024-09-08

**Authors:** Barbara Bayer, Shuting Liu, Katherine Louie, Trent R Northen, Michael Wagner, Holger Daims, Craig A Carlson, Alyson E Santoro

**Affiliations:** Division of Microbial Ecology, Centre for Microbiology and Environmental Systems Science, University of Vienna, Djerassiplatz 1, 1030 Vienna, Austria; Department of Ecology, Evolution and Marine Biology, Marine Science Institute, University of California, Santa Barbara, Lagoon Road, Santa Barbara, CA 93106, United States; Department of Ecology, Evolution and Marine Biology, Marine Science Institute, University of California, Santa Barbara, Lagoon Road, Santa Barbara, CA 93106, United States; Department of Environmental & Sustainability Sciences*,*Kean University, 1000 Morris Avenue, Union, NJ 07083, United States; Environmental Genomics and Systems Biology Division and DOE Joint Genome Institute, Lawrence Berkeley National Laboratory, Berkeley, CA 94720, United States; Environmental Genomics and Systems Biology Division and DOE Joint Genome Institute, Lawrence Berkeley National Laboratory, Berkeley, CA 94720, United States; Division of Microbial Ecology, Centre for Microbiology and Environmental Systems Science, University of Vienna, Djerassiplatz 1, 1030 Vienna, Austria; Department of Chemistry and Bioscience, Center for Microbial Communities, Fredrik Bajers Vej 7H, Aalborg University, 9220 Aalborg, Denmark; Division of Microbial Ecology, Centre for Microbiology and Environmental Systems Science, University of Vienna, Djerassiplatz 1, 1030 Vienna, Austria; The Comammox Research Platform, University of Vienna, Djerassiplatz 1, 1030 Vienna, Austria; Department of Ecology, Evolution and Marine Biology, Marine Science Institute, University of California, Santa Barbara, Lagoon Road, Santa Barbara, CA 93106, United States; Department of Ecology, Evolution and Marine Biology, Marine Science Institute, University of California, Santa Barbara, Lagoon Road, Santa Barbara, CA 93106, United States

**Keywords:** metabolomics, transcriptomics, Nitrosopumilus, Nitrospina, microbial interactions, nitrifier metabolite release, chemoautotroph-heterotroph associations

## Abstract

Microbial chemoautotroph-heterotroph interactions may play a pivotal role in the cycling of carbon in the deep ocean, reminiscent of phytoplankton-heterotroph associations in surface waters. Nitrifiers are the most abundant chemoautotrophs in the global ocean, yet very little is known about nitrifier metabolite production, release, and transfer to heterotrophic microbial communities. To elucidate which organic compounds are released by nitrifiers and potentially available to heterotrophs, we characterized the exo- and endometabolomes of the ammonia-oxidizing archaeon *Nitrosopumilus adriaticus* CCS1 and the nitrite-oxidizing bacterium *Nitrospina gracilis* Nb-211. Nitrifier endometabolome composition was not a good predictor of exometabolite availability, indicating that metabolites were predominately released by mechanisms other than cell death/lysis. Although both nitrifiers released labile organic compounds, *N. adriaticus* preferentially released amino acids, particularly glycine, suggesting that its cell membranes might be more permeable to small, hydrophobic amino acids. We further initiated co-culture systems between each nitrifier and a heterotrophic alphaproteobacterium, and compared exometabolite and transcript patterns of nitrifiers grown axenically to those in co-culture. In particular, B vitamins exhibited dynamic production and consumption patterns in nitrifier-heterotroph co-cultures. We observed an increased production of vitamin B_2_ and the vitamin B_12_ lower ligand dimethylbenzimidazole by *N. adriaticus* and *N. gracilis*, respectively. In contrast, the heterotroph likely produced vitamin B_5_ in co-culture with both nitrifiers and consumed the vitamin B_7_ precursor dethiobiotin when grown with *N. gracilis*. Our results indicate that B vitamins and their precursors could play a particularly important role in governing specific metabolic interactions between nitrifiers and heterotrophic microbes in the ocean.

## Introduction

Oceanic dissolved organic carbon (DOC) represents a large reservoir of reduced carbon in the ocean, nearly equal in size to the inorganic carbon reservoir in the atmosphere [1]. It consists of a complex mixture of compounds with turnover times ranging from minutes to thousands of years [2]. Consequently, the marine DOC pool plays a critical role in the long-term storage of carbon and, at the same time, represents the primary source of substrates for heterotrophic marine microbes. The transfer of organic carbon from phytoplankton to bacteria via a pool of labile dissolved compounds is a key process in the oceanic carbon cycle [3, 4]. It is estimated that photoautotrophs release up to 40% of net primary production into the marine DOC pool, supporting between 2 and 50% of the heterotrophic carbon demand in the surface ocean [5]. However, DOC production is not restricted to photoautotrophs and appears to be a widespread feature among marine microbes including heterotrophs [5] and chemoautotrophs [6].

The release of organic compounds by chemoautotrophs might represent an important source of DOC available to heterotrophic food webs in the deep ocean, reminiscent of organic carbon transfer between photoautotrophs and heterotrophs in the surface ocean. Nitrifiers, including ammonia- and nitrite-oxidizing microorganisms, are the most abundant chemoautotrophs in most parts of the global ocean [7]. In particular, ammonia-oxidizing archaea (AOA) constitute a considerable fraction of microbial biomass in the ocean [8], comprising up to 40% of the microbial communities in the deep ocean [9]. Very recently, DOC release by marine nitrifiers was quantified under multiple culture conditions across phylogenetically diverse taxa, suggesting that AOA and nitrite-oxidizing bacteria (NOB) release between 5 and 15% of their recently fixed inorganic carbon into the surrounding seawater [10]. AOA have previously been shown to release amino acids, vitamins, and other labile components of oceanic dissolved organic matter (DOM) [6]. However, the composition of DOC released by marine NOB remains largely uncharacterized. Although DOC released by nitrifiers is predicted to contribute only a small fraction (<1%) to the heterotrophic microbial carbon demand [6, 10], the release of physiologically important metabolites might be critical for microbes that are auxotrophic for these compounds.

Labile organic carbon release from autotrophs to surrounding microbes can occur by multiple mechanisms, including diffusion [11], active release for nutrient acquisition and communication [12, 13], carbon overflow for energy dissipation [14], and cell death from processes such as protist grazing and viral lysis [15, 16]. Passive diffusion of intracellular metabolite pools into external seawater is constrained to molecules of relatively small size [11, 17], which has been suggested to represent an important mechanism for the release of small amino acids in AOA [6]. Active release of metabolites can also occur in response to the presence of other microbes, which has been explored in phytoplankton-bacteria interactions [18–20]. Yet, there is a general lack of understanding of how metabolite composition and release in nitrifiers are affected by the presence and potential interactions with heterotrophic bacteria [21].

To explore links between nitrifying chemoautotrophs and heterotrophs via extracellular release of labile metabolites, we established two different model communities either containing the AOA *Nitrosopumilus adriaticus* CCS1 or the NOB *Nitrospina gracilis* Nb-211 co-cultured with the alphaproteobacterium *Qipengyuania citrea* H150. The latter was isolated from a nitrifier enrichment culture from the California Current system, which contained both ammonia-oxidizing archaea and nitrite-oxidizing *Nitrospinaceae* bacteria [22]. Although *Nitrosopumilus* and *Nitrospina* are typically genera with low abundance in the dark ocean [8, 23], their core metabolism and many genomic adaptations are shared with abundant, but as-of-yet uncultured nitrifiers in the deep ocean [24, 25]. Consequently, the simple co-culture communities selected here might represent relevant chemoautotroph-heterotroph associations and can thus give insights into the role of the largely unconstrained flux of DOC from chemoautotrophs in the dark ocean.

## Material and methods

### Nitrifier cultivation and enumeration

*Nitrosopumilus* sp. CCS1 was isolated from the California Current system in the North Pacific Ocean [10]. Its complete genome shares ~98% average nucleotide identity with the published genome of *Nitrosopumilus adriaticus* NF5 isolated from the Adriatic Sea [26, 27]. Hence, we refer to “*Nitrosopumilus* sp. CCS1” as “*Nitrosopumilus adriaticus* CCS1” throughout this manuscript. In contrast to strain NF5, strain CCS1 can grow in artificial seawater without additions of organic HEPES buffer [10], resolving previous issues with downstream metabolite extraction and quantification [6]. *N. gracilis* Nb-211 was originally isolated from surface waters of the South Atlantic Ocean by Watson and Waterbury [28] and its genome has recently been sequenced [29]. Both nitrifiers were grown axenically in an artificial seawater medium as previously described [30]. Culture medium of both nitrifiers was supplemented with 0.5 mM NH_4_Cl and catalase (2–5 U mL^−1^, Sigma-Aldrich) to reduce oxidative stress [31]. Additionally, 1 mM NaNO_2_ and 50 ng L^−1^ cyanocobalamin (Sigma-Aldrich) were added to *N. gracilis* Nb-211. Nitrifiers were grown in 1 L or 2 L glass bottles (Schott) and incubated at 25°C in the dark without agitation. Nitrite (NO_2_^−^) concentrations were measured using the Griess-Ilosvay colorimetric method [32], and cells were enumerated on an Easy-Cyte 5HT flow cytometer (Millipore Guava) following SYBR Green staining, as previously described [30].

The purity of the cultures was regularly checked via microscopy and by checking for heterotrophic growth after incubation in liquid marine broth (Difco 2216). The absence of predators was confirmed by microscopy, following DAPI staining of cells on 0.2 μm polycarbonate membranes (GTTP, Millipore). To investigate the presence of viruses, samples were prepared following the procedures described in [33], and particles counted on a CytoFLEX flow cytometer (Beckman Coulter). Although there was no evidence for abundant viral particles in nitrifier cultures, viruses with small genomes might not be sufficiently detected with this method [34] and their presence cannot be excluded.

### Heterotroph cultivation and enumeration

We isolated a heterotrophic alphaproteobacterium from a nitrifier enrichment culture from the California Current system [22]. According to recent re-classifications of members of the *Erythrobacteraceae* family [35], the heterotrophic isolate is referred to as *Qipengyuania citrea* H150 (originally *Erythrobacter citreus*). Axenic cultures of *Q. citrea* were obtained by plating on Marine Agar (Bacto 2216) and incubating at 18°C. Liquid cultures were grown in an artificial seawater medium, containing 1 mM acetate, 0.5 mM NH_4_Cl, 1 mM NaNO_2_, 50 ng L^−1^ cyanocobalamin, and catalase (2–5 U ml ^−1^).

### Establishment of nitrifier-heterotroph co-cultures

Nitrifier and heterotroph cultures were grown individually as described above. Before establishing co-cultures, *Q. citrea* H150 cells were collected from 10 ml culture via centrifugation (5000 × *g*, 10 min, 20°C), washed three times, and resuspended in artificial seawater medium. To prevent the cultures from becoming inactive, nitrifiers were not subjected to centrifugation. Cell densities of nitrifiers and washed *Q. citrea* H150 cells were determined via flow cytometry as indicated above. Each nitrifier was grown in triplicate both axenically and in co-culture with the heterotroph. Inoculum sizes to initiate cultures were 10% for *N. adriaticus* CCS1 and 3.5% for *N. gracilis* Nb-211, yielding starting cell concentrations of ~5 × 10^6^ ml ^−1^ for *N. adriaticus* CCS1 and ~10^6^ ml ^−1^ for *N. gracilis* Nb-211. The higher initial cell concentrations of *N. adriaticus* compared to *N. gracilis* co-cultures were chosen to achieve similar growth patterns (similar length of growth curve and cell yields at the end of incubation). The heterotroph was added at cell concentrations of ~10^5^ ml ^−1^.

**Fig. 1:**
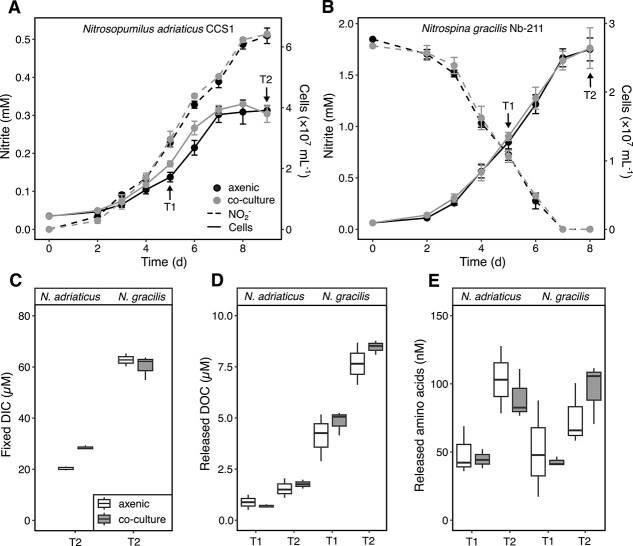
Growth of the ammonia-oxidizing archaeon *Nitrosopumilus adriaticus* CCS1 (**A**) and the nitrite-oxidizing bacterium *Nitrospina gracilis* Nb-211 (**B**) when grown axenically or in co-culture with a heterotrophic bacterium. An increase in nitrite concentrations in *N. adriaticus* cultures indicates ammonia oxidation to nitrite and a decrease in nitrite concentrations in *N. gracilis* cultures indicates nitrite oxidation to nitrate. The mean of three biological replicates is shown for each measurement and error bars depict the standard deviation. Further reported are concentrations of fixed dissolved inorganic carbon (DIC) (**C**), carbon released as DOC (**D**), and released amino acids (**E**) during growth (T1: Exponential growth phase, T2: Stationary growth phase). Box plots show the median and the first and third quartiles (boxes) as well as the minimum and maximum values (whiskers). Experiments to identify potential differences in growth and activity parameters between treatments were exploratory, and we did not set out to test specific hypotheses before conducting these experiments. We therefore refrain from doing any statistical analyses on hypotheses generated after analysis of these data.

NO_2_^−^ concentrations and cell abundances were monitored at regular intervals (see Fig. 1A,B). Cell enumeration of *Q. citrea* in co-cultures was impeded as their visualization partly overlapped with nitrifiers in flow cytograms. In an attempt to overcome this issue, we plated co-cultures on marine agar throughout the entire incubation period to determine how many viable *Q. citrea* cells were present. After 3 days of incubation, yellow circular colonies formed on agar plates, which could be enumerated manually. However, heterotroph cell abundances counted on plates showed a high variability potentially due to cell aggregation, resulting in uneven cell distribution on agar plates. Hence, cell abundances of the heterotroph in co-cultures (Fig. S1) should be taken as an approximation.

### Metabolite extraction and metabolome analyses

All plastic and glassware used were rinsed with acidified ultrapure water (Milli-Q, HCl analytical grade, Sigma-Aldrich, pH 2), and glassware was combusted at 500°C for 5 h. Cultures were grown in 1 or 2 L glass bottles as described above, and samples for metabolomics were taken during the early stationary growth phase.

For exometabolomes, culture supernatants of 1 L of culture were obtained via vacuum filtration through 0.2 μm pore size PES membranes (47 mm diameter, Millipore). DOM was extracted from culture supernatants and uninoculated culture media by solid phase extraction (SPE) as previously described [36]. Briefly, supernatants were acidified to pH 2 and extracted on Bond Elut PPL sorbent SPE cartridges (200 mg, Agilent). Subsequently, cartridges were rinsed with 2 × 3 ml acidified (pH 2) ultrapure water, air-dried, and eluted with 3 ml methanol into amber glass vials. Procedural blanks were prepared by processing culture medium in the same way. Extracts were dried down in an oven (45°C) for 4–5 h and vials were stored at −20°C until analyses.

Cellular endometabolomes were obtained by filtering 1 L of culture and subsequently washing cells from filters by gently resuspending them in 1 ml of artificial seawater medium. Cells were centrifuged down (5000 × *g*, 10 min, 20°C) and cell pellets were frozen and lyophilized dry (FreeZone 2.5 Plus, Labconco), then powderized by bead-beating (2 × 5 sec, BioSpec MiniBeadbeater96). To extract metabolites, 400 μl of 100% methanol was added, then samples vortexed and sonicated for 10 min in a water bath, centrifuged (2350 × *g*, 5 min, 20°C) to pellet cell debris, and the supernatant dried in a SpeedVac (Spd111V, Thermo Scientific) and stored at −80°C until analysis.

Dried PPL exometabolome and pellet endometabolome extracts were resuspended in 120 μl of 100% methanol, centrifuge-filtered (0.22 μm PVDF membrane, Millipore) and transferred to glass LC–MS vials. LC–MS analysis was performed using an Agilent 1290 UHPLC stack coupled to a Thermo Orbitrap QExactive HF mass spectrometer (Thermo Scientific) using normal-phase liquid chromatography, as described previously [37]. Samples were run in both positive and negative ionization mode, with metabolites identified using custom MetAtlas software [38] based on comparing retention time, mass/charge ratio, and fragmentation spectra to a database of compound standards run in-house using the same LC–MS/MS methods. Additionally, internal isotopically labeled standards of amino acids and other organic compounds were added to a subset of samples during methanol resuspension. Only metabolites present in all three biological replicates, which had peak intensities 5 times those of media blanks, were considered.

### Dissolved organic carbon and free amino acid quantification

DOC concentrations were measured by high-temperature combustion using a modified Shimadzu TOC-V following the procedures described in [39]. DOC concentrations of the solid-phase extracted DOM were quantified as previously described [40]. Extraction efficiencies of DOC on PPL cartridges were calculated from initial DOC concentrations and by taking into account the concentration factor of DOC by SPE.

For the analysis of extracellular dissolved free amino acids (DFAA), 1 ml of culture was filtered through 0.1 μm syringe filters with PVDF membrane (Millex, Millipore), and the filtrate was stored in 1.5 ml combusted amber glass vials at −20°C until analysis. DFAA were analyzed using a Thermo Scientific high-performance liquid chromatography (HPLC) system (ICS-5000+) equipped with a fluorescence detector (FLD-3000). DFAA were pre-column derivatized with *o*-phthaldialdehyde, separated on a C18 column (Acclaim 120, 5 μm, 120 Å, 4.6 × 250 mm) and measured following an established gradient program [41–43]. Amino acid concentrations of medium blanks were subtracted from sample concentrations.

### Measurements of dissolved inorganic carbon fixation and DOC release

Ten μCi [^14^C]-bicarbonate (specific activity 56 mCi mmol^−1^/2.072 × 10^9^ Bq mmol^−1^, Perkin Elmer) was added to 10 ml of culture. For every culture condition, at least three replicate live samples and one dead control were incubated in temperature-controlled incubators in the dark, and incubations were terminated when cultures reached stationary growth phase by adding formaldehyde (3% v/v). Dissolved inorganic carbon (DIC) fixation and DOC release rates were measured as described previously [10].

### RNA extraction and transcriptome analyses

During exponential and stationary growth phases, cells were harvested by filtering 500 ml of culture through 0.2 μm pore size PES membranes (47 mm diameter, Millipore). RNA was extracted according to [44] with modifications for the use of filters as previously described [31]. Complete DNA removal was validated via PCR amplification of 16S rRNA gene sequences during 30 cycles and quality of RNA was checked with Bioanalyzer RNA chip profiles (Agilent Technologies).

Transcriptome sequencing was completed at the DOE Joint Genome Institute (JGI) using the NovaSeq system (Illumina; JGI SOP 1065.1). Reads were trimmed, quality-filtered, and contaminants were removed (see details in the *Supplementary Information*). The remaining filtered reads were mapped to the complete genomes of *Nitrosopumilus adriaticus* CCS1*, N. gracilis* Nb-211, and *Qipengyuania citrea* H150 and quantified using HTSeq [45]. Transcript counts (transcripts per million, TPM) for each sequenced library are reported in Table S1.

Differential levels of expression between treatments (axenic, co-culture) and growth stages (exponential, stationary) were tested separately for each species with the DESeq2 Bioconductor package (version 1.34.0) [46] in the R software environment (version 4.1.2) [47] using transcript counts as input data. A Wald test was used for hypothesis testing as implemented in the DESeq2 package, and *P* values were adjusted using the Benjamini-Hochberg correction method [46]. Transcripts that showed significant pairwise correlations (adjusted *P* value <0.05; ≥2-fold change between treatments) were visualized with the pheatmap package (version 1.0.12) [48]. DESeq2 results are reported in Table S2.

## Results and discussion

### Co-culture growth dynamics and DOC release

This study was designed to enhance our understanding of metabolite release and utilization in two nitrifier-heterotroph co-culture systems. The extracellular release of metabolites derived from either *N. adriaticus* CCS1 or *N. gracilis* Nb-211 was the sole source of organic carbon available for the heterotrophic bacterium *Qipengyuania citrea* H150. We explored the growth and activity dynamics of each species either grown axenically or in nitrifier-heterotroph co-cultures.

At the start of the incubation, *N. adriaticus* CCS1 and *N. gracilis* made up 97 ± 1% and 86 ± 3% of cells in nitrifier-heterotroph co-cultures, respectively. The high contribution of nitrifiers in co-cultures was chosen to ensure the growth of the heterotroph despite the low cell-specific DOC release rates of *N. adriaticus* CCS1 and *N. gracilis* [10]. Throughout the growth curve, nitrifier cell abundance ratios oscillated between 67 and 94% in AOA-heterotroph co-cultures and between 52 and 90% in NOB-heterotroph co-cultures. *N. adriaticus* CCS1 appeared to have slightly faster growth rates when grown in co-culture compared to axenic cultures (Fig. 1). Although it was not possible to completely distinguish between nitrifier and *Q. citrea* cells in flow cytograms (see *Material and Methods*), evidence from viable plate counts suggests that cell abundances of the heterotroph were relatively stable between days 3–6 (Fig. S1). Furthermore, the magnitude of DIC fixation by *N. adriaticus* CCS1 was ~1.4 times higher in co-cultures compared to axenic cultures during stationary phase (Fig. 1), which further suggests a positive effect of the heterotroph on AOA growth yields and DIC fixation. Although we cannot exclude a contribution of the heterotroph to anaplerotic DIC fixation, a lack of increase in cell abundance of *Q. citrea* from day 3 onwards (Fig. S1) suggests a low metabolic activity and, consequently, a low contribution to DIC fixation. In contrast, growth rates and DIC fixation yields of *N. gracilis* remained unaffected by the presence of the heterotroph (Fig. 1). In a previous study, three *Nitrosopumilus* species, including the closely related strain *N. adriaticus* NF5, showed no increased growth response in co-culture with the heterotrophic alphaproteobacterium *Oceanicaulis alexandrii* [31]. This suggests that metabolic interactions between nitrifiers and heterotrophs might be species-specific, as has been observed in phytoplankton-heterotroph associations [19, 49, 50]. When grown in co-culture with both nitrifiers, *Q. citrea* exhibited double sigmoid growth patterns with an increase in cell abundance during the first 3–4 days and towards the end of incubation after 8–9 days (Fig. S1). We assume that the initial increase in cell abundance might have been supported by metabolites present in the nitrifier culture inocula. There was no clear difference between the cell yields of the heterotroph in co-culture with either *N. adriaticus* CCS1 or *N. gracilis* (Fig. S1).

*N. adriaticus* CCS1 released ~2 μM DOC by stationary phase (Fig. 1D), which represented ~9% and ~6% of fixed DIC in axenic cultures and when grown in co-culture, respectively. *N. gracilis* released ~8 μM DOC by stationary phase making up ~12% of fixed DIC during both culture conditions. Although nitrifier DOC release remained relatively consistent between treatments, DOC uptake by the heterotroph in co-cultures is not accounted for in our measurements, thus possibly underestimating the magnitude of DOC release in co-cultures. Cell-normalized DOC release rates were higher in *N. gracilis* (~40 amol cell^−1^ d^−1^) compared to *N. adriaticus* CCS1 (~5 amol cell^−1^ d^−1^), which can be explained by higher per-cell DIC fixation rates and generally larger cell sizes of *Nitrospina* bacteria compared to AOA [6, 23, 51]. Amino acids made up ~6% and ~1% of the released DOC by stationary phase in *N. adriaticus* CCS1 and *N. gracilis*, respectively, indicating differences in the composition of the DOM released between the two nitrifiers.

### Intracellular metabolite composition

We characterized the intracellular metabolite compositions of the two nitrifiers *N. adriaticus* CCS1 and *N. gracilis* Nb-211 during early stationary growth. The endometabolome reflects the pool of metabolites that might be released to the surrounding water via diverse mechanisms, including diffusion, active release, or cell death due to viral lysis or predation.

**Fig. 2:**
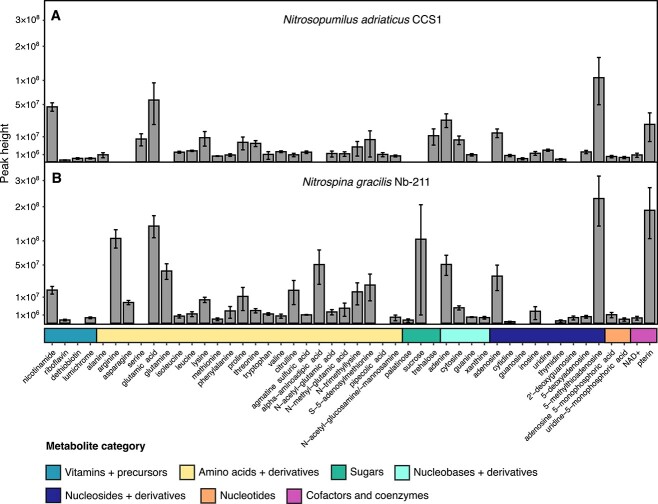
Endometabolomes of *Nitrosopumilus adriaticus* CCS1 (**A**) and *Nitrospina gracilis* Nb-211 (**B**). Peak heights are square-root transformed to increase readability. Only endometabolites with peak heights at least 5 times higher than procedural blanks are shown. The mean of three biological replicates is shown for each metabolite and error bars depict the standard deviation. The prediction of palatinose is somewhat uncertain (level 1 prediction) and the identified peak could correspond to a different disaccharide. Peak heights of metabolites might not reflect absolute metabolite abundances and should rather be used as a proxy for their abundance.

The majority of the detected endometabolites in both nitrifiers consisted of amino acids, nucleobases, nucleosides, and their respective derivatives (Fig. 2), yet metabolite abundance patterns differed between *N. adriaticus* and *N. gracilis*. Although arginine and glutamine were two of the most abundant amino acids detected in *N. gracilis* cells, they were not detectable above media blanks in the endometabolomes of *N. adriaticus*. In contrast, serine was detected only in *N. adriaticus* cells (Fig. 2). The endometabolomes of both nitrifiers contained relatively high amounts of 5-methylthioadenosine and pterin. The former is an important intermediate in the biosynthesis of spermidine/spermine from S-adenosylmethionine and putrescine. However, S-adenosylmethionine carboxylase, which catalyzes the first step in the metabolic pathway [52], is missing from the genomes of both *N. adriaticus* CCS1 and *N. gracilis*. Alternatively, *N. adriaticus* CCS1 does encode the complete pathway for diphthamide biosynthesis, which post-translationally modifies histidine residues in translation elongation factor 2, producing 5-methylthioadenosine in the first step of the pathway [53]. Pterin was particularly abundant in endometabolomes of *N. gracilis*. Pterin derivatives are common cofactors including folates and molybdopterin [54]. Elevated levels of pterin found in cells of *N. gracilis* may suggest a high requirement for the molybdoprotein nitrite oxidoreductase (NXR) enzyme, which is responsible for catalyzing the first step in the main energy-conserving pathway in the cell, and typically represents a significant portion of the proteome in nitrite oxidizers (~13 500 NXR copies cell^−1^, [30]).

Organic osmolytes are small metabolites typically present at high intracellular concentrations (mM) in many marine microbes to regulate their osmotic balance [55]. *Nitrosopumilus maritimus* SCM1 has been shown to produce the osmolytes ectoine and hydroxyectoine [56], yet only few AOA species encode the biosynthetic gene cluster for ectoine/hydroxyectoine production, and no alternative osmolytes have been identified yet [26, 57, 58]. We detected trehalose in the endometabolomes of *N. adriaticus* CCS1 (Fig. 2A), an osmolyte commonly synthesized by diverse marine microbes [55], suggesting its role in maintaining the osmotic balance in AOA cells. However, known trehalose biosynthesis pathways are missing in the genomes of AOA including *N. adriaticus* CCS1. Marine NOB encode genes for the biosynthesis of diverse osmolytes including glycine betaine, (hydroxy-)ectoine, trehalose, and sucrose [30, 51]. We detected the latter in high relative amounts in *N. gracilis* cells (Fig. 2B) concurrent with the presence of a complete sucrose biosynthesis pathway in its genome, suggesting that sucrose might be an important osmolyte in some members of the *Nitrospinaceae* family.

### Metabolite release and utilization patterns

We identified ecologically relevant exometabolites in axenic nitrifier cultures and in nitrifier-heterotroph co-culture systems, in which DOM released by *N. adriaticus* CCS1 or *N. gracilis* served as the sole carbon source for a bacterial heterotroph. The exometabolite pool was characterized when nitrifiers reached early stationary growth and no measurable cell decay has yet occurred. In the absence of predators and no detectable viruses in our culture systems (see *Material and Methods*), exometabolome analysis therefore pinpoints metabolites released via diffusion or active transport. Exometabolome samples require desalting and concentration of metabolites before mass spectrometry, which results in significant losses of material escaping analytical characterization. DOC extraction efficiencies across all samples were 31 ± 13%, which was higher than observed in phytoplankton cultures [59], but lower compared to average extraction efficiencies of oceanic DOC [60]. To mitigate some of the analytical bias of metabolite extraction, we also quantified and characterized the low molecular weight DFAA in exometabolomes via conventional HPLC methods (see *Material and Methods*). Additionally, metabolite release could be influenced by environmental conditions, which was not directly addressed in our study. Yet, substrate concentration and temperature were shown to not affect the quantity of released DOC in nitrifier cultures [10].

**Fig. 3:**
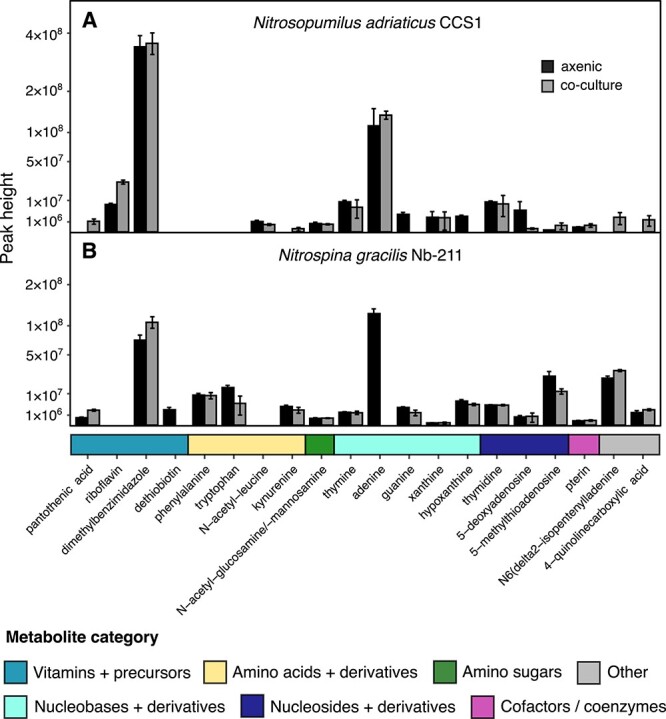
Exometabolomes of *Nitrosopumilus adriaticus* CCS1 (**A**) and *Nitrospina gracilis* Nb-211 (**B**). Exometabolite peak heights of axenic cultures are shown in black and those of nitrifier-heterotroph co-cultures in grey. Peak heights are square-root transformed to increase readability. Only exometabolites with peak heights at least 5 times higher than those of culture media blanks are shown. The means of three biological replicates is shown for each metabolite and error bars depict the standard deviation. Due to minor differences in cell abundances, peak heights of the same metabolites can be compared between axenic vs. co-cultures of the same nitrifier strain, yet peak heights of different metabolites cannot be compared directly and should rather be used as proxy for their abundance. Experiments to identify potential differences in metabolite abundances between treatments were exploratory, and we did not set out to test specific hypotheses before conducting these experiments. We therefore refrain from doing any statistical analyses on hypotheses generated after analysis of these data.

Nitrifier exometabolomes consisted of vitamin and vitamin precursors, amino acids, nucleobases, nucleosides, and their derivatives (Fig. 3). Two of the dominant exometabolites from both nitrifiers were dimethylbenzimidazole (DMB) and adenine. DMB is a lower (alpha) ligand of cobalamin (vitamin B_12_) and is produced from riboflavin (vitamin B_2_), which was detected in endometabolomes of both nitrifiers (Fig. 2) and the exometabolomes of other *Nitrosopumilus* species [6]. Cobalamin has previously been suggested as an important metabolic currency between AOA and other microbes [61, 62], however, most marine NOB, including *N. gracilis*, are reported to be auxotrophic for vitamin B_12_ [30, 51, 63, 64]. Although the complete cobalamin biosynthesis pathway is missing in *N. gracilis* [29], the genes required for the biosynthesis of DMB are present. The release of DMB by vitamin B_12_ auxotrophs highlights unexpected dynamics of vitamin precursor exchange between microbial community members. The availability and cycling of B_12_ lower ligands in the ocean has been recognized to potentially play a more significant role than previously assumed [65, 66]. Nucleobases and nucleosides can be released as a result of a salvage bottleneck due to the absence of central enzymes [67, 68], which has previously been suggested for thymidine release in other *Nitrosopumilus* species [6]. However, both nitrifiers encode the complete adenine salvage pathway suggesting that the release of adenine is unlikely to result from an incomplete pathway.

**Fig. 4:**
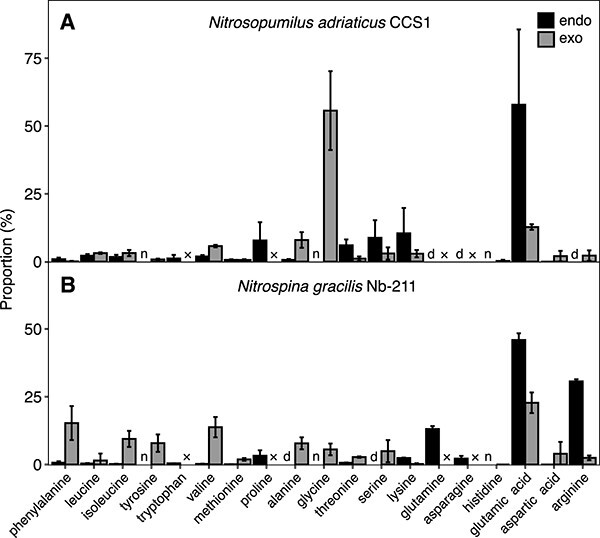
Dissolved free amino acid (DFAA) composition in endo- and exometabolomes of *Nitrosopumilus adriaticus* CCS1 (**A**) and *Nitrospina gracilis* Nb-211 (**B**). DFAA concentrations and composition in the exometabolomes were measured via HPLC (see *Material and Methods* section). Values are calculated as the percentage of total DFAAs in exometabolomes (based on concentrations) and endometabolomes (based on peak heights) (×, not measured; n, not detected; d, detected but <5 times blank concentrations). Tryptophan was not measured via HPLC but has been detected in exometabolomes (see Fig. 3). Glutamine and asparagine are esterified after derivatization and appear as glutamic acid and aspartic acid in exometabolome samples. Amino acids are sorted based on their hydrophobicity [73] from left to right, starting with the most hydrophobic amino acid phenylalanine. It should be considered that different hydrophobicity scales are available and glycine is either referred to as neutral or hydrophobic amino acid. The means of three biological replicates is shown for each amino acid and error bars depict the standard deviation.

Amino acids represent important cellular metabolites and favorable substrates for heterotrophic microbes in the ocean [69–72]. Glycine was the dominant amino acid in exometabolomes of *N. adriaticus* CCS1, making up ~55% of all measured amino acids (Fig. 4A), which has previously been observed in three *Nitrosopumilus* species [6]. Additionally, alanine, glutamic acid and valine made up ~6–13% each, whereas other amino acids were only detected at very low concentrations. The exometabolomes of *N. gracilis* were dominated by glutamic acid (~23%), phenylalanine (~15%), and valine (~14%), and contained lower amounts (~8–9%) of alanine, isoleucine and tyrosine (Fig. 4B). In contrast, endometabolomes of both nitrifiers were dominated by glutamic acid (~50%), whereas arginine additionally made up a high fraction (~30%) in endometabolomes of *N. gracilis*. Ammonia is typically incorporated into biomolecules through glutamate and thus, glutamate/glutamic acid is present at elevated concentrations in most cells. Additionally, glutamate can also serve as an osmolyte in marine microbes [55]. Hydrophobic amino acids [73] have previously been suggested to be preferentially released by AOA via passive diffusion [6], potentially due to the higher permeability of the lipid bilayer to hydrophobic compared to hydrophilic amino acids [74]. Furthermore, it has recently been proposed that archaeal lipid membranes might be more permeable than bacterial membranes, particularly to small amino acids such as alanine and glycine [75]. The preferential release of small and/or hydrophobic amino acids in archaea is consistent with the ~6-times higher proportion of amino acids relative to total released DOC in *N. adriaticus* CCS1 compared to *N. gracilis* (Fig. 1D,E). However, *N. adriaticus* CCS1 and *N. gracilis* expressed a putative amino acid/polyamine transporter and an ABC amino acid transport system, respectively (Table S1), leaving open the possibility of active amino acid import/export from the cell. Albeit expressed at very low relative abundances, these could potentially facilitate (selective) re-uptake of amino acids.

We additionally compared exometabolome signatures between axenic *N. adriaticus* CCS1 and *N. gracilis* cultures to those grown in co-culture with the heterotroph. Nitrifier-derived exometabolites that were depleted in the co-culture media compared to axenic cultures were putatively identified as compounds that were consumed by the heterotroph. For example, the peak heights of adenine and tryptophan decreased ~9- and ~3-fold in co-culture compared to axenic *N. gracilis* cultures, respectively, whereas dethiobiotin and guanine disappeared completely from the culture medium (Fig. 3B). Dethiobiotin is an important precursor of biotin (vitamin B_7_), which is an essential cofactor for carboxyl group transfer enzymes such as acetyl-CoA carboxylase [76]. In contrast, only very few detected metabolites showed different peak heights in axenic *N. adriaticus* cultures compared to co-cultures (Fig. 3A), except guanine, which decreased ~3-fold in *N. adriaticus*-heterotroph co-cultures. In contrast, some vitamins and vitamin precursors accumulated in co-cultures compared to axenic nitrifier cultures, either suggesting production by the heterotrophic bacterium or an influence of the heterotroph on the synthesis and release by the nitrifiers. Pantothenic acid (vitamin B_5_) increased ~4–6-times in co-cultures of both nitrifiers compared to axenic cultures (Fig. 3). Additionally, riboflavin increased ~3-fold and DMB ~1.5-fold in *N. adriaticus* and *N. gracilis*-heterotroph co-cultures, respectively. None of the three species have been reported to be auxotrophic for pantothenic acid or riboflavin. However, *Q. citrea* does not possess genes for cobalamin biosynthesis, including for DMB. This implies that *N. gracilis* may have increased the production and/or release of DMB in the presence of *Q. citrea* despite the inability of the heterotroph to use this compound for cobalamin biosynthesis.

### Transcriptional response of nitrifiers in co-culture with a heterotroph

We examined concurrent transcript inventories of both nitrifiers during exponential and stationary growth in axenic cultures and in co-culture with the heterotroph *Q. citrea.* Due to the extremely low number of mapped transcripts (<50 000) obtained from *Q. citrea* grown in co-culture, we were unable to reliably quantify changes in transcript abundances indicative of metabolite consumption by the heterotroph. This low read recruitment was likely due to the over-representation of nitrifiers in co-cultures, the lack of growth and low metabolic activity of *Q. citrea*, and the presence of an antisense RNA [77] which made up ~80% of all mapped reads in *Q. citrea* transcriptomes. We focused instead on analyzing the transcriptional response of each nitrifier to the presence of the heterotroph, and during different growth phases.

The largest differences in transcript abundance were observed between exponential and stationary phase nitrifier cultures, with ~600 and ~1500 genes differentially expressed between exponential and stationary phase in axenic *N. adriaticus* CCS1 and *N. gracilis*, respectively (Table S2). Growth-phase-dependent differences of transcript abundances in *N. adriaticus* CCS1 were higher compared to differences observed in AOA cultures upon ammonia limitation [78, 79]. In contrast, only 87 genes were differentially expressed in *N. adriaticus* co-cultures compared to axenic cultures during exponential phase and 384 were differentially expressed between the two treatments in stationary phase (Table S2). In *N. gracilis* co-cultures, 55 genes were differentially expressed compared to axenic cultures during both growth stages. Although *N. gracilis* showed the same growth patterns in axenic cultures and co-cultures (Fig. 1B), the higher growth rates in *N. adriaticus* CC1 co-cultures compared to axenic cultures could potentially affect transcription rates, making direct comparisons between treatments more difficult. Consequently, we focused on genes that exhibited the largest changes in abundance independent of growth stage.

**Fig. 5:**
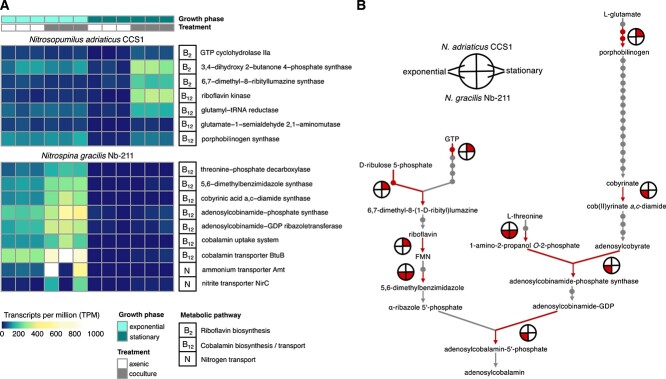
(**A**) Transcript abundances of nitrifier genes that demonstrated significantly higher expression (adj. *P* value <0.05; ≥2-fold change) in co-cultures with a heterotroph compared to axenic cultures (see *Material and Methods* section). Differential expression analyses of all genes can be found in the *supplementary information* (Table S2). (**B**) Expression of key enzymes in cobalamin and riboflavin biosynthesis pathways are shown. Red lines indicate steps of the pathway were genes showed higher expression in co-culture compared to axenic nitrifier cultures and large circles indicate for which nitrifier in which growth phase these observations were made (see legend). Every small dot represents an intermediate compound in the pathway.

Some of the most pronounced changes in transcript abundances in both nitrifiers in response to the heterotroph were related to vitamin B biosynthesis (Fig. 5). *N. adriaticus* CCS1 expressed genes for the complete riboflavin and cobalamin biosynthesis pathways, however, only a few of these genes were differentially expressed in co-culture (Table S2). These included genes for the first reactions of riboflavin biosynthesis (Fig. 5), suggesting that the higher amounts of riboflavin detected in exometabolomes of AOA-heterotroph co-cultures (Fig. 3A) might be released by *N. adriaticus* CCS1*.* Furthermore, genes involved in the synthesis of cofactor FMN from riboflavin and the biosynthesis of the cobalamin precursor porphobilinogen were more highly expressed in co-culture compared to axenic *N. adriaticus* cultures during stationary growth (Fig. 5). The higher expression of genes at several entrance points to the cobalamin biosynthesis pathway suggests a requirement for a higher flux of intermediates to upregulate the synthesis of this important vitamin. As indicated by its gene content, the co-cultured heterotroph is auxotrophic for cobalamin and must meet its requirements from cobalamin produced by *N. adriaticus* CCS1. In contrast, both partners are auxotrophic for cobalamin in NOB-heterotroph co-cultures, though *N. gracilis* encodes genes for several steps of the pathway. These include all required genes for the biosynthesis of precorrin-2 and sirohydrochlorin, as well as for the production of adenosylcobalamin from the precursor cobyrinate. *N. gracilis* increased the expression of some of these genes in response to the heterotroph, including genes involved in the synthesis of cob(II)yrinate a,c-diamide from cobyrinate, and at the branching points of adenosylcobinamide-phosphate and of adenosyl-5′-phosphate synthesis (Fig. 5B). Furthermore, 5,6-DMB synthase showed higher expression, corroborating the higher amounts of DMB detected in co-cultures compared to axenic *N. gracilis* cultures (Fig. 3D). As cyanocobalamin was added to *N. gracilis* culture medium, the expression of genes involved in intermediate steps of the cobalamin pathway was unexpected and may reflect a lack of gene regulation. We hypothesize that *N. gracilis* may continuously produce and release cobalamin precursors to attract potential partners, which could provide the required additional steps of the pathway. At the same time, cobalamin uptake systems were detected at higher abundances in response to the heterotroph (Fig. 5A).

In addition to genes involved in vitamin biosynthesis and transport, *N. gracilis* also increased the expression of ammonium and nitrite transporters during exponential growth in co-cultures compared to axenic cultures (Fig. 5A). Because nitrite oxidoreductase faces the periplasmic side in *Nitrospinaceae* [63], nitrite transport is not required for nitrite oxidation. Consequently, the higher expression of both ammonium and nitrite transporters by *N. gracilis* indicates competition for nitrogen for assimilatory purposes during co-culture conditions. In contrast, genes for ammonium transport and nitrogen regulatory protein P-II were among the most transcribed genes by the heterotroph *Q. citrea* in co-culture with *N. adriaticus* CCS1 during stationary growth (~80 times higher compared to *N. gracilis* co-cultures; Table S2), suggesting that *N. adriaticus* CCS1 outcompeted the heterotroph for ammonium. Marine AOA have been shown to have exceptionally high specific affinities for ammonia/ammonium [80, 81], providing them with a competitive advantage to successfully compete for reduced nitrogen with heterotrophic bacteria and phytoplankton.

## Conclusions

Interactions between nitrifiers and heterotrophic bacteria in the deep ocean are reminiscent of phytoplankton-bacteria interactions in surface waters, representing carbon transfer reactions relevant at a global scale. We compared and contrasted changes in the metabolome composition and expression patterns in axenic nitrifier cultures and nitrifier-heterotroph co-cultures. Nitrifier exo- and endometabolomes consisted of many labile organic carbon compounds, which could fuel bacterial heterotrophy (i.e., amino acids, sugars) and/or supplement auxotrophies (i.e., for B vitamins). Our results highlight that nitrifier endometabolome composition was not a good predictor of exometabolite availability, suggesting that metabolite release mechanisms such as passive diffusion and active transport are more important compared to cell death/lysis as previously reported for ammonia-oxidizing archaea [6]. Furthermore, detected metabolites could not always be linked to biosynthesis pathways, such as trehalose in *N. adriaticus* CCS1 cells, for which corresponding biosynthesis genes are missing from its genome. The larger nitrite oxidizer *N. gracilis* had higher DOC release rates per cell compared to the ammonia oxidizer *N. adriaticus*, which preferentially released amino acids, corroborating previous observations that archaeal membranes might be more permeable to small amino acids than bacterial membranes [75].

Nitrifier transcription patterns were dynamic, strongly differed with the growth stage, and were affected by the presence of the heterotroph. Particularly, B vitamins showed dynamic production and consumption patterns in co-culture compared to axenic nitrifier cultures, which were accompanied by higher transcription levels of vitamin B biosynthesis genes (Fig. 5). *N. adriaticus* and *N. gracilis* likely increased the production of riboflavin (vitamin B_2_) and the vitamin B_12_ lower ligand DMB in response to the heterotroph, respectively, whereas the heterotroph produced vitamin B_5_ (pantothenic acid) in co-culture with both nitrifiers and consumed the vitamin B_7_ precursor dethiobiotin when grown with *N. gracilis.* These results suggest that B vitamins and their precursors might play an important role in governing specific metabolic interactions between nitrifiers and heterotrophic microbes in the ocean, and that these complex physiological interactions underlie metabolite exchange. An improved understanding of the factors that influence nitrifier metabolite release and their successive consumption by heterotrophic microbes will be essential to untangling this important but nearly invisible carbon flux.

## Supplementary Material

SI_Metabolomics_Nitrifiers_Manuscript_BB_240508_wrae172

TableS1_wrae172

TableS2_wrae172

## Data Availability

The genomes of *Nitrosopumilus adriaticus* CCS1, *N. gracilis* Nb-211, and *Qipengyuania citrea* H150 are available in the GenBank database under accession numbers CP167059.1, GCA_021845525.1, and GCA_024171405.1, respectively. Raw transcriptome sequencing data generated during the current study are available in the NCBI sequence read archive (SRA) repository under accession numbers SRP500926-SRP500962. Metabolomics raw data generated during the current study are available in the MassIVE data repository (https://massive.ucsd.edu/), accession number MSV000094763. Microbial strains reported in the manuscript are available from the authors due to the fastidious nature of nitrifiers and associated difficulties with depositing them to culture collections. All other data generated or analyzed during this study are available in the manuscript or its associated *Supplementary Information*.
