# Supplementary material for: Metabolite release by nitrifiers facilitates metabolic interactions in the ocean
Source: ISME J. 2024 Sep 8;18(1):wrae172. doi: 10.1093/ismejo/wrae172 (PMC11428151; doi:10.1093/ismejo/wrae172)
Supplement: SI_Metabolomics_Nitrifiers_Manuscript_BB_240508_wrae172 [file si_metabolomics_nitrifiers_manuscript_bb_240508_wrae172.pdf]

Supplementary Information

Metabolite release by nitrifiers facilitates metabolic interactions in the ocean

Barbara Bayer[1,2]*, Shuting Liu[2,3], Katherine Louie[4], Trent R. Northen[4], Michael Wagner[1,5], Holger Daims[1,6], Craig. A. Carlson[2], and Alyson E. Santoro[2]

[1] Centre for Microbiology and Environmental Systems Science, Department of Microbiology and Ecosystem Science, University of Vienna, Vienna, Austria

[2] Department of Ecology, Evolution and Marine Biology, University of California, Santa Barbara, CA, USA

[3] Department of Environmental & Sustainability Sciences, Kean University, Union, NJ, USA

[4] DOE Joint Genome Institute, Lawrence Berkeley National Laboratory, Berkeley, CA, USA

[5] Center for Microbial Communities, Department of Chemistry and Bioscience, Aalborg University, Aalborg, Denmark

[6] The Comammox Research Platform, University of Vienna, Vienna, Austria

*Correspondence: Barbara Bayer, barbara.bayer@univie.ac.at

Supplementary Methods

Transcriptome sequencing and analysis

Transcriptome sequencing was completed at the DOE Joint Genome Institute (JGI) using Illumina technology (JGI SOP 1065.1). Briefly, rRNA was depleted from an input of 100 ng of total RNA using QIAseq FastSelect™ – 5S/16S/23S, rRNA Plant and rRNA Yeast Kits (Qiagen). Using the TruSeq stranded mRNA kit (Illumina), the 300 bp - 400 bp heat fragmented RNA was reverse transcribed to create the first strand of cDNA with random hexamers and SuperScript™ II Reverse Transcriptase (Thermo Fisher Scientific) followed by second strand synthesis. The double stranded cDNA fragments were treated with A-tailing, ligation with JGI's unique dual indexed adapters (IDT) and enriched using 10 cycles of PCR. The prepared libraries were quantified using KAPA Biosystems' next-generation sequencing library qPCR kit and run on a Roche LightCycler 480 real-time PCR instrument. Sequencing of the flowcell was performed on the Illumina NovaSeq sequencer using NovaSeq XP V1.5 reagent kits, S4 flowcell, following a 2x151 indexed run recipe. BBDuk (version 38.96) (https://jgi.doe.gov/data-and-tools/software-tools/bbtools/) was used to remove contaminants using the following parameters: trim reads that contained adapter sequence and homopolymers of G's of size 5 or more at the ends of the reads, right quality trim reads where quality drops below 6, remove reads containing 1 or more 'N' bases, remove reads with average quality score across the read less than 10, having minimum length <= 49 bp or 33% of the full read length. Reads mapped with BBMap (https://jgi.doe.gov/data-and-tools/software-tools/bbtools/) to human, cat, dog and mouse references at 93% identity were removed. Additionally, reads aligned to common microbial contaminants (JGI SOP 1077) were also removed. Remaining filtered reads were mapped to the complete genomes of *Nitrosopumilus* sp. CCS1, *Nitrospina gracilis* Nb-211 and *Qipengyuania citrea* H150 available on IMG/JGI and quantified using HTSeq (Anders *et al.* 2015).

References:

Bushnell B: BBTools software package, https://bbtools.jgi.doe.gov

Anders S, Pyl PT, Huber W. HTSeq—a Python framework to work with high-throughput sequencing data. *Bioinformatics* 2015; 31: 166-69; https://doi.org/10.1093/bioinformatics/btu638

Supplementary tables and figures

Table S1. Transcriptome counts (transcripts per kilobase million, TPM)

Table S2. Differential expression analyses (DESeq2 analyses results)

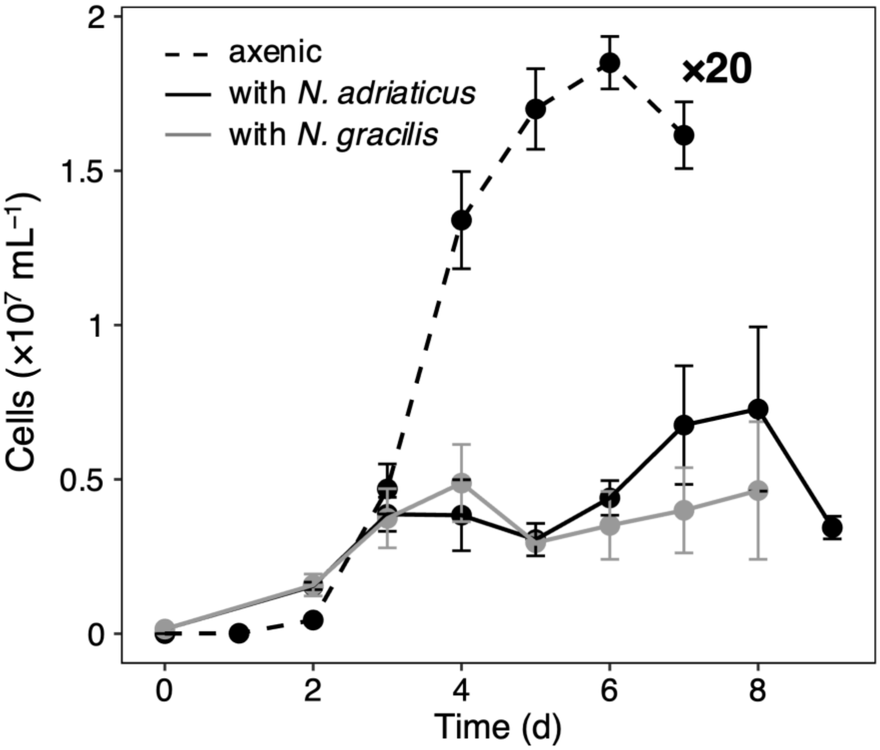

Figure S1. Growth dynamics of the heterotrophic alphaproteobacterium *Qipengyuania citrea* H150 either grown axenically on acetate (dashed black lines) or in co-culture with the ammonia-oxidizing archaeon *Nitrosopumilus adriaticus* CCS1 (solid black lines) or the nitrite-oxidizing bacterium *Nitrospina gracilis* Nb-211 (solid grey lines) without addition of organic carbon compounds. Note that cell abundances of axenically grown cultures were divided by a factor of 20 to fit growth curves on the same scale. The mean of three biological replicates is shown for each measurement and error bars depict the standard deviation.
